# Hydatid cyst of thyroid gland, a rare case report with a literature review

**DOI:** 10.1016/j.ijscr.2020.02.019

**Published:** 2020-02-11

**Authors:** Abdwlwahid M. Salih, Zanyar Y. Abdulla, Dlawar A. Mohammed, Vanya I. Jwamer, Pshtiwan G. Ali, Ahmed G. Hamasaeed, Hawar H. Shkur, Jalal K. Omer, Rawezh Q. Salih, Shvan H. Mohammed, Aso S. Muhialdeen, Karzan Mohammed, Snur Othman, Fahmi H. Kakamad

**Affiliations:** a College of Medicine, Department of Surgery, University of Sulaimani, François Mitterrand Street, Sulaimani, Kurdistan, Iraq; b Sulaimaniy Polytechnical University Presidency, Sulaimani, Kurdistan, Iraq; c Shahid Dr. Kassem Main Health Center, Directorate General of Health Germian, Sulamani, Kurdistan, Iraq; d College of Medicine, University of Sulaimani, François Mitterrand Street, Sulaimani, Kurdistan, Iraq; e College of Pharmacy, University of Sulaimani, François Mitterrand Street, Sulaimani, Kurdistan, Iraq; f Takya Main Health Center, Sub Directorate of Health Chamchamal, Kurdistan, Iraq; g Blood Bank Center, Sulaimani, Kurdistan, Iraq; h Kscien Organization for Scientific Research, Hamdi Street, Sulaimani, Kurdistan, Iraq; i Iraqi Board For Medical Specialties, Sulaimani Teaching Hospital, Sulaimani, Kurdistan, Iraq; j Faculty of Medical Sciences/School of Medicine, Department of Cardiothoracic and Vascular Surgery, University of Sulaimani, François Mitterrand Street, Sulaimani, Kurdistan, Iraq; k Islamic University of Madinah, Madinah, Saudi Arabia

**Keywords:** Echinococcus, Multilocularis, Granulosus, Anaphylaxis

## Abstract

**Introduction:**

Although hydatid cysts can affect any organ in the body, reports regarding affection of thyroid gland are scanty in the literature. This report aims to present a case of thyroid hydatid disease with literature review.

**Case report:**

A 48-year-old female presented with painless anterior neck mass of about 2 year duration. There was an ill-defined, central anterior neck mass, with a smooth surface and mobile with deglutition. Ultrasound of the thyroid gland revealed an enlarged left lobe of thyroid gland due to well defined thick wall cystic nodule. The patient underwent left thyroid lobectomy under general anesthesia, the pathology report revealed hydatid cyst of thyroid gland.

**Discussion:**

Liver and lungs are often the end destination for hydatid cysts, while other places like mediastinum, diaphragm, cardiac, smooth and skeletal muscles, abdominal and chest walls are rarely involved. In this case, the cyst affected even a rarer organ which was the left lobe of thyroid gland. Most of the time the disease is asymptomatic and is found accidentally, yet depending on the site and size of the cysts, symptoms can occur.

**Conclusion:**

Hydatid disease of thyroid gland is an extremely rare condition. The main presenting symptom is swelling. Operation under general anesthesia is the only modality of treatment.

## Highlights

• Hydatid disease is an infestation by Echinococci species in the endemic areas.
• Liver and lungs are the main two affected organs.
• Hydatid disease of thyroid is an extreme rare variant of the condition
• In this report, the details of thyroid hydatid disease occurring in a middle age female with brief literature review

## 1 Introduction

Hydatidosis as a zoonotic disease represents one of the oldest known diseases, dating back to Hippocrates era [1]. It is caused by Echinococcus multilocularis and Echinococcus granulosus infestation [2] from the Taeniidae family in the Cestode class [3]. Due to travelling, the disease is seen worldwide [1], however it is most endemic in areas with mild climates like Australia, Southeast Asia, New Zealand, Middle East, Mediterranean countries and South America [4]. Up to two-third of the affected patients present with liver involvement, and up to 25% present with lung involvement, while other organs involvement like bone, kidney, muscle, pancreas, heart and brain constitute a small portion [5]. However even in endemic areas head and neck hydatidosis is a rare finding [6]. When mistook for malignancy, needle aspiration can endanger the patient’s life [7]. In line with SCARE guideline, we present a case of hydatid cyst in the thyroid gland, along with a brief literature review [8].

### 1.1 Patient information

A 48-year-old female patient presented with a painless anterior neck mass of about 2 year duration. The mass increased in size slowly and associated with mild shortness of breath. The patient was living in a village, working on a farm and she has a long history of animal contact such as sheep, goats and dogs. Past surgical history was positive for plastic operation for a burned face.

### 1.2 Clinical findings

On examination; there was an ill-defined, central anterior neck mass, with a smooth surface and mobile with deglutition. Investigations including complete blood counts, thyroid function tests, renal function tests, blood sugar and chest-X-ray were normal. Anti-thyroid peroxidase (anti-TPO) antibodies were negative. Ultrasound of the thyroid gland revealed an enlarged left lobe of thyroid gland (65 × 33 × 32 mm) due to a well-defined thick wall (reaching 4 mm) cystic nodule measuring about 40 × 32 × 32 mm, occupying the mid-low third of left lobe, with a mild peripheral vascularity and microcalcification.

### 1.3 Therapeutic intervention

**Fig. 1:**
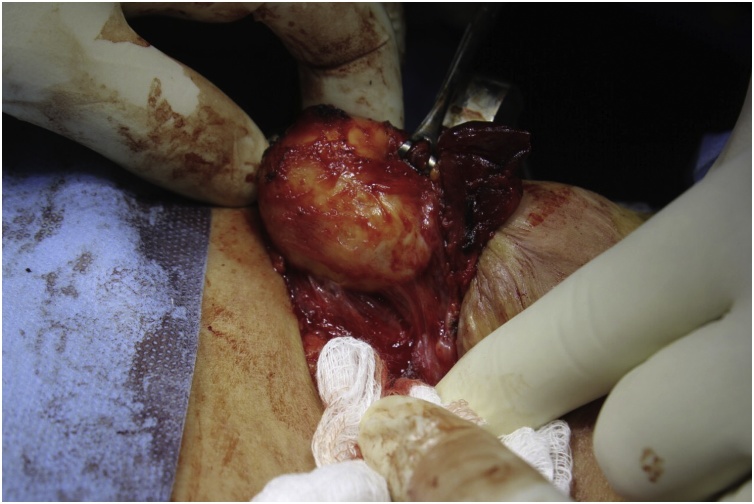
intraoperative picture showing whitish cyst consistent with cyst’s germinal layers.

The patient was prepared for general anesthesia (GA). Under GA, she underwent left thyroid lobectomy. The operation was completed within one hour. the pathology report revealed hydatid cyst of thyroid gland with pericystic adhesion (Fig. 1).

### 1.4 Follow-up and outcomes

Postoperatively, the patient was sent for computed tomography scan (CT scan) of the head, neck, chest, abdomen and pelvis, the result was negative. The patient was remained in hospital for two days. The patient was put on anthelminthics for three months. Six months later, the patient was free from recurrence.

## 2 Discussion

Hydatid disease is a parasitic infection seen all over the world, though mostly in regions like Eastern Europe, South Africa, Middle East, South America, Australia and Mediterranean region where cattle and sheep rearing is common [2]. The current case has history of sheep and cattle contact.

Liver and lungs are often the end destination for cysts, while other places like mediastinum, diaphragm, cardiac, smooth and skeletal muscles, abdominal and chest walls [[9], [10], [11], [12], [13], [14]] are rarely involved. In this case, the cyst affected even a rarer organ which was the left lobe of thyroid gland. Just like this case, it is seen more frequently among middle-aged women [5]. Most of the time, the disease is asymptomatic and is found accidentally, yet depending on the site and size of the cysts, symptoms can occur. Hewa et al. received a 22-year-old with complete paraplegia, later proven to be Hydatid disease of spine and recovered after operation [15]. This patient presented with painless anterior neck mass of about 2 year duration.

Echinococci species pass through three developmental stages in their life cycle, in the adult form they reside in their definitive host intestines and these are carnivores, most commonly dogs. Then their eggs will be excreted to the environment through feces and when ingested by intermediate hosts, which commonly include pig, camel, cattle, goats and sheep, they form metacestodes [10]. Humans are not naturally included in their biological life cycle, instead may accidentally become an intermediate host when ingesting food contaminated by dog feces containing the echinococcosis eggs [16,17]. Once the eggs reach the gastrointestinal system, they will rupture and larvae emerge. These larvae penetrate the intestinal wall and reach the hepatic sinusoids through the portal system. Larvae that reach the lung are small enough to escape liver’s filtration system [3].

Ultrasonography (USG), Computed Tomography scan (CT), Magnetic Resonance Imaging (MRI), Scintigraphy and fine-needle aspiration cytology (FNAC) are the standard methods for diagnosing Hydatid cyst [3]. To this day worldwide, complete surgical excision with perioperative albendazole is most commonly practiced and regard as the standard. The results are outstanding with low recurrence rate.

Two forms of thyroid gland hydatid cysts have been described [2,3]. When the diagnosed hydatid disease is confined to the thyroid gland with no evidence of the disease elsewhere, this is referred to as primary form. The literature contains very few reports of the primary form of thyroid hydatid disease (Table 1). Presence of other organ involvement like liver, lung and others labels the disease as secondary form [3]. This case is primary thyroid hydatid cyst due to not having the cyst before or elsewhere. When the size of the cyst increases, it may mimic thyroid carcinoma and adhere to surrounding structures like strap muscles, recurrent laryngeal nerve, trachea, esophagus and carotid sheath [2]. In this case, the cyst was surrounded by normal thyroid tissue of the left lobe.

**Table 1:** brief review of the reported cases of thyroid hydatid disease.

Author/Reference	Year	Number of cases	Age	Sex	Presenting Symptoms	Management
Eshraghi et al./ [26]	2019	1	34 years	Female	growing tangible swelling in her neck (left side)	left lobectomy and isthmusectomy
Oksuz et al./ [29]	2013	1	23 years	Male	hoarseness of voice and left lobe mass	subtotal thyroidectomy
Danilă et al./ [23]	2015	1	26 years	Female	nodular goiter with moderate compression symptoms.	total thyroidectomy
Hoysal et al./ [27]	2019	1	14 years	Female	swelling in front of her neck (left side)	left hemithyroidectomy
Bastanhagh et al./ [7]	1995	3	16,24,60	Female	lump in her neck (Right), (Lift) and (Right) respectively	Rt lobectomy, excision, excision
Dey et al./ [25]	2014	1	30 years	Female	neck lump Right side	Albendazole (conservative treatment)
Jain et al./ [24]	2005	1	55 years	Female	midline neck mass	excision
Azendour et al./ [2]	2011	1	23 years	Female	enlarging neck mass, Right side	subtotal thyroidectomy
Rajabian et al./ [30]	1991	2	16,27	Female	right thyroid nodule, right thyroid nodule	excision, excision
Jiang et al./ [28]	2019	1	54 years	Male	gradual swelling on the left side of the lower part of the neck	excision
Söğütlü et al./ [32]	2007	1	18 years	Male	swelling of the thyroid gland left side	hemi-thyroidectomy with isthmusectomy
Yesim Erbil, MD/ [21]	2005	2	21,70 years	Male, Female	swelling in the left lobe, right solitary thyroid nodule	left hemithyroidectomy, bilateral near-total thyroidectomy
Akbulut et al./ [3]	2015	2	26,57 years	Female	neck swelling, hoarseness, and neck swelling	total thyroidectomy, total thyroidectomy
Saha et al./ [30]	2007	1	30 years	Male	right sided thyroid swelling	patient refused surgery
Ozdemir et al./ [1]	1989	1	54 years	Female	nodular goiter	right total and left subtotal thyroidectomy
Avcu et al./ [6]	2010	1	48 years	Male	right side neck lump	Albendazole, and aspiration
Sultana et al./ [19]	2016	1	35 years	Female	swelling in neck	excision
Batrin et al./ [2]	2015	1	32 years	Female	palpable and growing mass in the neck right side	bilateral total thyroidectomy
Lada et al./ [34]	2005	1	28-year	Male	left cervical tumefaction	lobectomy with isthmusectomy
Amahzoune et al./ [33]	2004	1	21-year	Male	cold thyroid nodule	Resection

Currently medical treatments are not effective, thus surgical excision still provides the best outcome. Calò et al. resected hydatid cyst of the trapezius in an old age male under local anesthesia [18]. Medical treatment with albendazole or mebendazole may still be tried in cases unfit for surgery, although adverse reactions and unpredictable results have been reported.

When performing surgery, the aim is to remove the cyst completely while avoiding spillage of its content. Reported complication include organ damage, abscess and secondary cyst formation and most importantly anaphylaxis if the cyst ruptures [19,20,22,31]. The current case was free from complication.

## 3 Conclusion

Hydatid disease of thyroid gland is an extremely rare condition. The main presenting symptom is swelling. The diagnosis is suspected by ultrasound while it only can be confirmed after pathological examination of the specimen. Operation under general anesthesia is the only modality of treatment.

## Sources of funding

No source to be stated.

## Ethical approval

Approval is not necessary for case report in our locality.

## Consent

Consent has been taken from the patient and the family of the patient.

## Author contribution

Abdulwahid M. Salih: Surgeon performed the operation and follow up.

Shvan H. Mohammed, Fahmi H. Kakamad, and Rawezh Q. Salih: Writing the manuscript and follow up.

:literature review, final approval of the manuscript.

## Registration of research studies

Not applicable.

## Guarantor

Fahmi Hussein kakamad.

## Provenance and peer review

Not commissioned, externally peer-reviewed.

## Declaration of Competing Interest

Here is no conflict to be declared.
